# Evaluation and feasibility of diagnostic heatflow imaging in patients with palpable breast lesions: a pilot study

**DOI:** 10.1007/s00404-025-08093-5

**Published:** 2025-06-28

**Authors:** Patrik Pöschke, Axel Boese, Katharina Seitz, Niklas Amann, Sophie Eckstein, Carla E. Schulmeyer, Carolin C. Hack, Felix Heindl, Hanna Huebner, Andreas Füller, Peter A. Fasching, Julius Emons

**Affiliations:** 1 Department of Gynecology and Obstetrics, Comprehensive Cancer Center Erlangen EMN (CCC-ER-EMN), Bavarian Cancer Research Center (BZKF), Erlangen University Hospital, Friedrich-Alexander-Universität Erlangen-Nürnberg (FAU), Universitätsklinikum ErlangenUniversitaetsstrasse 21–23, 91054 Erlangen, Germany; 2 Comprehensive Cancer Center Erlangen-EMN (CCC ER-EMN), Erlangen, Germany; 3 Comprehensive Cancer Center Alliance WERA (CCC WERA), Erlangen, Germany; 4 Bavarian Cancer Research Center (BZKF), Erlangen, Germany; 5 INKA Innovation Laboratory for Image Guided Therapy, Otto-Von-Guericke University Magdeburg, 39120 Magdeburg, Germany

**Keywords:** Breast cancer, Thermographic imaging, Heat flow imaging (HFI)

## Abstract

**Background:**

Breast cancer is the most common cancer in women, with early detection significantly improving outcomes. Heat flow imaging (HFI) is a non-invasive dynamic thermography method with prior tissue cooling. It has shown its potential as an additional diagnostic tool. The aim of the present study was to evaluate the feasibility of diagnostic HFI in patients with palpable breast lesions during outpatient visits.

**Methods:**

The patients presenting with palpable breast lesions at the Erlangen University Hospital were recruited between November 2023 and April 2024. Heat flow imaging was performed in addition to sonographic and mammographic imaging in routine care. Additionally, the patients completed a pain questionnaire to evaluate the comfort of the procedure. We used a two-phase study design. During the first phase, the imaging procedure was established and standardized. In the second phase, imaging footage was compared with conventional mammography, sonography, and histological findings.

**Results:**

Thirty-nine patients were recruited and 18 patients underwent final evaluation. Heat flow imaging successfully detected 7 out of 11 palpable carcinomas. Factors contributing to missed lesions and impairing image quality were inadequate cooling or improper camera positioning. The mean pain score reported during the procedure was 0.7 on a visual analog scale from 0 to 10, indicating minimal discomfort.

**Conclusions:**

Heat flow imaging is a feasible imaging method that may serve as a supplementary diagnostic tool for breast cancer detection in patients with palpable breast lesions. However, it is still considered an experimental method and its use should be limited in the context of clinical trials. Further research involving larger patient groups is required to validate these preliminary findings and to optimize image acquisition protocols.

**Supplementary Information:**

The online version contains supplementary material available at 10.1007/s00404-025-08093-5.

## What does this study add to the clinical work

**Table:** 

Heat flow imaging is a feasible supplementary diagnostic tool for routine outpatient evaluation of patients with palpable breast lumps. Further research is needed to optimize imaging protocols and validate its clinical utility.	

## Introduction

Breast cancer is the most frequent cancer in women, with one out of eight women developing this kind of cancer [1]. In the last decades, morbidity and mortality of breast cancer patients have been significantly improved by early cancer detection, primarily due to organized breast cancer screening and early cancer detection programs including screening mammography [2–11] and adjunctive imaging such as ultrasound [5–7, 12, 13], and magnetic resonance imaging (MRI) in selected patients [7, 14].

Currently German recommendations were updated and offer women who are 50 to now 75 years old, with average risk for breast cancer, a mammography every 2 years [15]. Even though the detection of breast cancer has improved, there are still tumors, which cannot be detected easily by mammography. Especially younger patients presenting with palpable breast lesions are not included in mammography screening programs. Tumors in mammographic dense breasts are overlooked on mammograms more often than tumors in low dense breasts [16, 17]. The risk factors masking influencing breast cancer detection on mammograms are patient’s age, body mass index, previous breast surgery and percent mammographic density (PMD) [18]. PMD is the strongest predictor of mammographic failure [18, 19]. Nevertheless in addition to asymptomatic women eligible for screening, there are symptomatic patients presenting with palpable breast lesions. In this patient cohort clinical breast examination (CBE) [2, 5, 8, 10, 11, 20–24], sonography and mammography and MRI are also relevant and standardized tools to diagnose breast lesions, but adding a supplementary diagnostic procedure could be a promising approach.

Thermography, a non-invasive imaging technique that detects infrared radiation emitted by the body, is gaining traction as a complementary method for breast cancer diagnosis. Recent studies have underscored thermography’s potential to identify temperature variations associated with tumor growth, providing a functional assessment of tissue metabolism and vascular activity. Small studies showed that thermographic imaging could detect 92.31% of breast cancer lesions [25]. Sensitivity and specificity were described in literature comparable to mammography in 132 patients with 81.6% and 57.8% compared to 80.5% and 73.2%, respectively [26]. In a multicentric study with 769 patients examined prior to breast biopsy, a sensitivity of thermographic imaging of 97% with a specificity of 14% was shown [27]. An extension of the pure infrared thermography is heat flow imaging (HFI) [28]. It includes a prior superficial tissue cooling that brings the temperature level of the tissue out of balance. A thermographic image recording over time can then be used to measure the dynamics of reheating.

The aim of our study was to evaluate the feasibility of using a thermographic imaging camera—still considered an experimental diagnostic tool—in combination with additional cooling, in the clinical routine for patients presenting with palpable breast lesions. In addition, we assessed patient comfort using a standardized pain questionnaire [29].

## Methods

### Patient selection

The patients were recruited from November 2023 to April 2024 at the University Breast Center for Franconia, University Hospital Erlangen. The patients were treated in accordance to standard clinical care. They were eligible for inclusion in the study if they were at least 18 years old and presented with a palpable breast lesions. Standardized breast diagnostic took place in all patients in routine and biopsies of all suspicious lesions were performed following the clinical standard procedure. All patients provided written, informed consent for the study procedures and the scientific use of data. The patients included in this trial were enrolled in the i-MODE-B (Imaging and Molecular Detection–Breast) study. The ethics committee of the Friedrich-Alexander University Erlangen-Nuernberg provided approval for the study (ref. numbers 2700 and 297 17 Bc). The patients were excluded if a breast biopsy was taken within the last 7 days before scheduled HFI.

### Clinical data

**Fig. 1:**
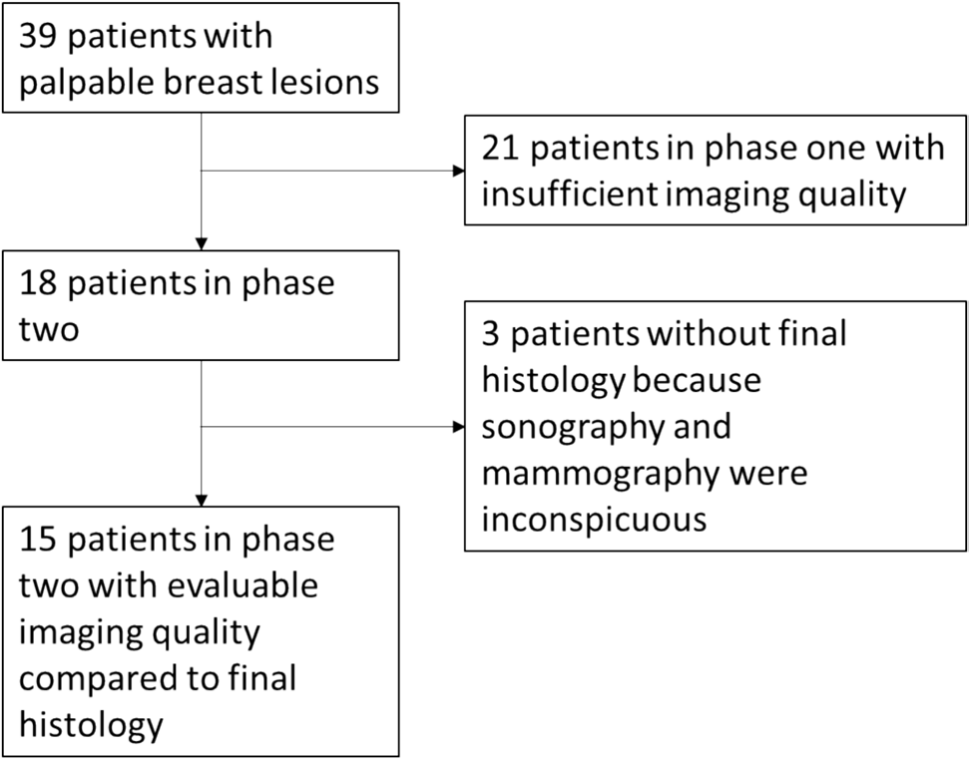
Patient selection

The University Breast Center for Franconia is a breast cancer center certified by the German Society for Breast Diseases (Deutsche Gesellschaft für Senologie) and the German Cancer Society (Deutsche Krebsgesellschaft). All clinical data were collected prospectively during clinical routine (see Table 1 and Fig. 1). Additionally, patients filled out a pain questionnaire (see Suppl. Table 1). Data management was facilitated using a Microsoft Access database 2019. Descriptive analysis was conducted using IBM SPSS Statistics for Windows, Version 28.0 (IBM Corp., Released 2021, Armonk, New York).

**Table 1:** Patient and tumor characteristics

		HFI (*n* = 39)
Age	Mean (SD)	53.54 (16.0)
Menopausal status	Premenopausal	17 (44.7)
	Postmenopausal	21 (55.3)
Tumor histology	No histology	7 (18.4)
	Benign	12 (31.6)
	DCIS	2 (0.5)
	Invasive breast cancer	19 (50.0)
Tumor stage	T1	7 (36.8)
	T2	10 (52.6)
	T3	2 (10.5)
	T4	0
Lymph node status	N0	11 (57.9)
	N +	8 (42.1)
Grading	G1	2 (10.5)
	G2	7 (36.7)
	G3	9 (47.4)
HER2 status	HER2-	16 (84.2)
	HER2 +	3 (15.8)
Estrogen receptor (ER) status	ER-	6 (31.6)
	ER +	13 (68.4)
Progesterone receptor (PR) status	PR-	6 (31.6)
	PR +	13 (68.4)

Mean (standard deviation, SD) where appropriate are shown for continuous characteristics and frequency (percentage) for categorical characteristics

*HFI* heat flow Imaging, *ER* estrogen receptor, *PR* progesterone receptor, *SD* standard deviation, *N* number, *DCIS* ductal carcinoma in situ, *HER2* human epidermal growth factor receptor 2

### Trial design

**Fig. 2:**
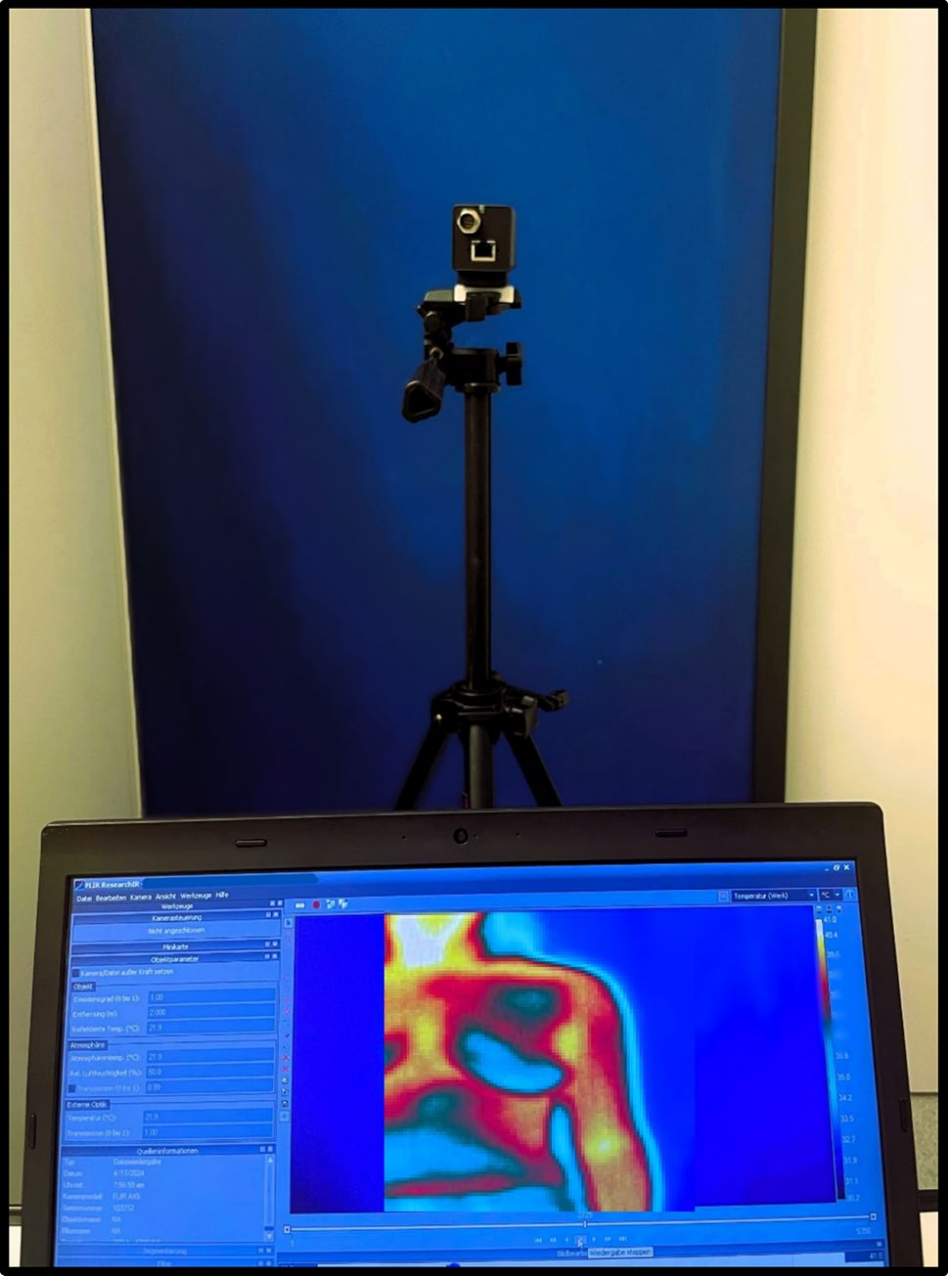
Standardized imaging setup

This study used a two-phase design. In the first phase, the focus was on establishing the HFI methodology. This included standardizing the imaging procedure, the cooling process, and the image acquisition setup (see Fig. 2). Figure 2 shows the examination set up as described above and with an image optimized by ChatGPT Version 4.0 for easier understanding. The protocols were developed to ensure consistent cooling of the breast tissue, using a cooling pad of specified size, temperature, and duration. Additionally, the setup for capturing thermographic images was refined to maintain a fixed distance and angle between the infrared camera and the patient, ensuring good diagnostic quality and reproducibility.

In the second phase, the standardized methodology was applied to a cohort of 18 patients presenting with palpable breast lumps during routine outpatient care. The thermographic images were obtained and analyzed according to those established protocols. The results were compared with mammographic and sonographic imaging and histological findings to evaluate the feasibility of HFI in a clinical setting. A pain questionnaire was used to evaluate the discomfort experienced during the procedure (see Suppl. Table 1).

Only patients from phase two were included in the final analysis.

### Heat flow imaging

Heat flow imaging (HFI) involves a prior cooling of the breast surface to disturb the thermal balance, followed by monitoring the dynamics of the re-heating process within the breast tissue using an infrared imaging camera. Cancerous tissue, due to higher perfusion and elevated energy metabolism, reheats more rapidly compared to normal tissue [28, 30, 31]. The procedure was integrated into routine consultations following clinical breast examination. In phase one of the study common issues affecting imaging quality were detected. Based on the learnings of phase one, an imaging protocol was adapted and optimized.

**Fig. 3:**
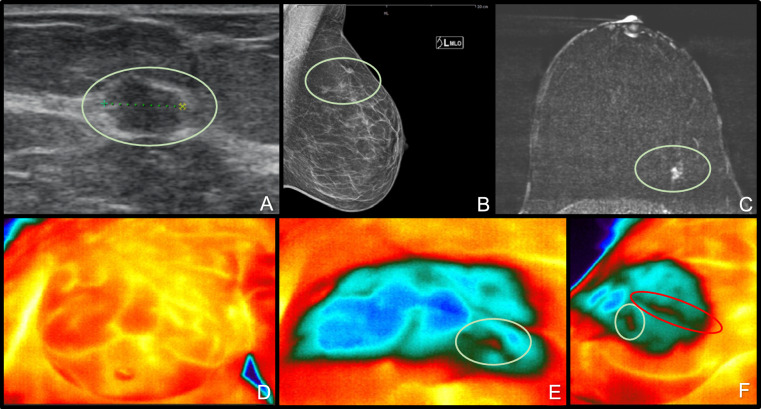
Top row left to right: Sonography (**A**), Mammography mediolateral-oblique view (**B**); Breast Computer Tomography (**C**); bottom row left to right: Heat flow imaging: native view (**D**), frontal view pad €, 45°angel view (**F**); the green circle show the carcinoma; the red circle a blood vessel

**Fig. 4:**
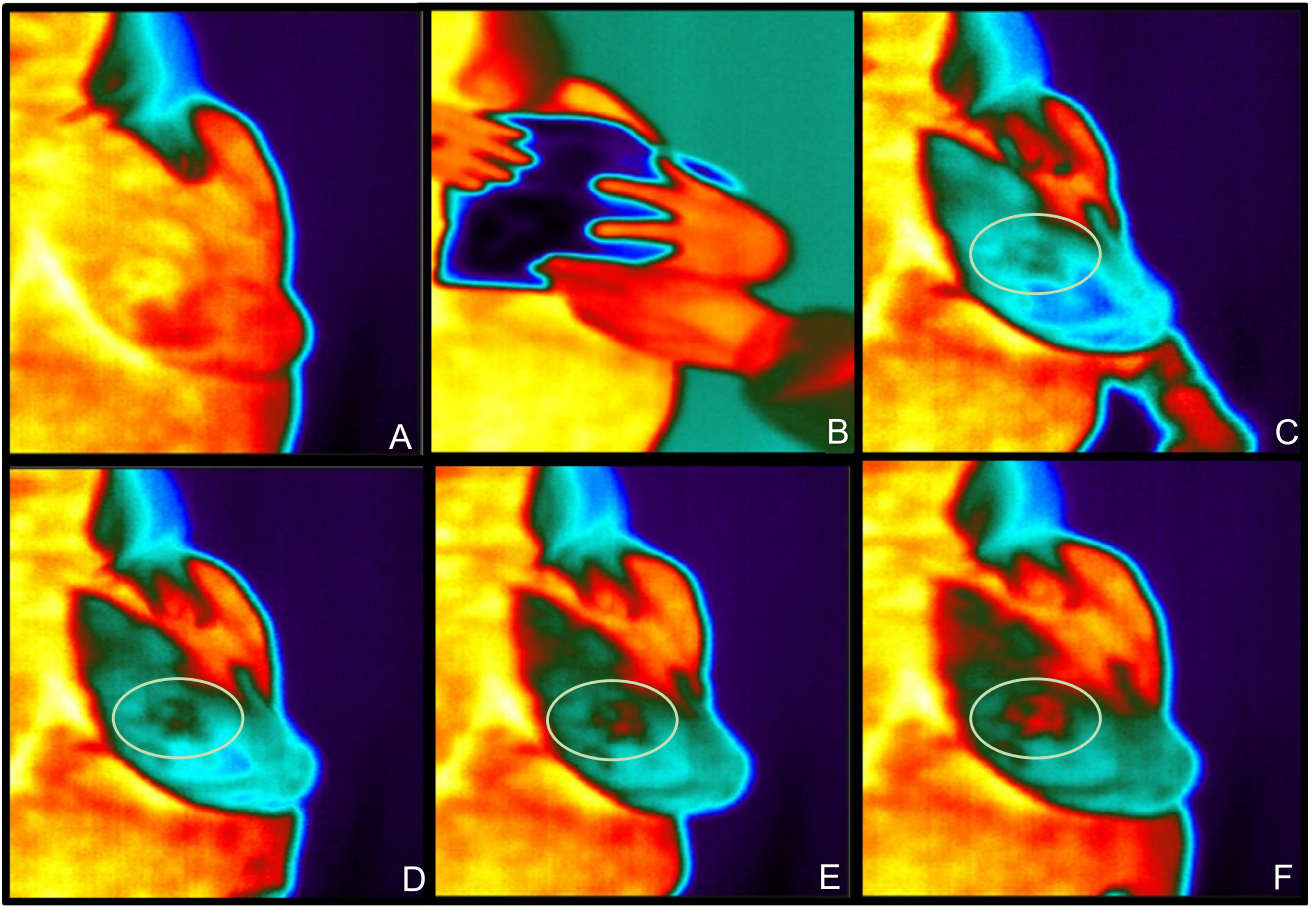
Top Row left to right and bottom row left to right: native view (**A**), cooling with pad (**C**), reheating after 10 s (**C**), reheating after 20 s (**D**), reheating after 30 s (**E**) and the final punctum maximum at 60 s reheating (**F**); the green circle is showing the carcinoma

During the established examination of phase two the optimized imaging protocol was used. The patients were positioned 50 cm from the IR camera, placed against a homogenous background. The optimal focus was maintained in real-time using the FLIR A65 camera and ResearchIR Version 4.40.12.38 (64 Bit) (March 14, 2022) software (FLIR Systems, Wilsonville, Oregon, United States) on a laptop. A 90-s video was recorded, which commenced with a 5-s baseline view. This was followed by a 25-s cooling phase using a cooling pad from a refrigerator set at 8 °C, and concluded with a 60-s re-heating period. The cooling pad measured 30 cm by 20 cm. After recording, the video was anonymized and saved for analysis (see Fig. 3 and Fig. 4).

### Patient questionnaire

The patients were asked to complete a pain questionnaire asking about any pain experienced and the comfort of the procedure, as well as any redness of the breast noticed after the examination (Suppl. Table 1). Finally, the patients were also asked to indicate their overall experience of pain using a visual analog scale from 0 to 10.

### Data evaluation

A physicist trained in the evaluation of thermographic images analyzed the images without knowledge of the histology or the lesion positions. Initially, a right-left comparison was conducted before cooling and used to identify superficial vessels and heat differences. Subsequently, immediately after cooling, the temperature map was evaluated with different levels, followed by observations of rewarming over time. The criteria for interpretation included the shape of rewarming anatomical structures, temperature differences, and temperature gradients. Based on these criteria, possible lesions were marked in the HFI. In a second step, these results were compared with the results of mammographic and sonographic imaging results as well as histology as gold standard. Mammographic imaging was evaluated according to BI-RADS® Breast Imaging Reporting & Data System; American College of Radiology; Reston, VA, USA) during clinical routine [32].

## Results

**Fig. 5:**
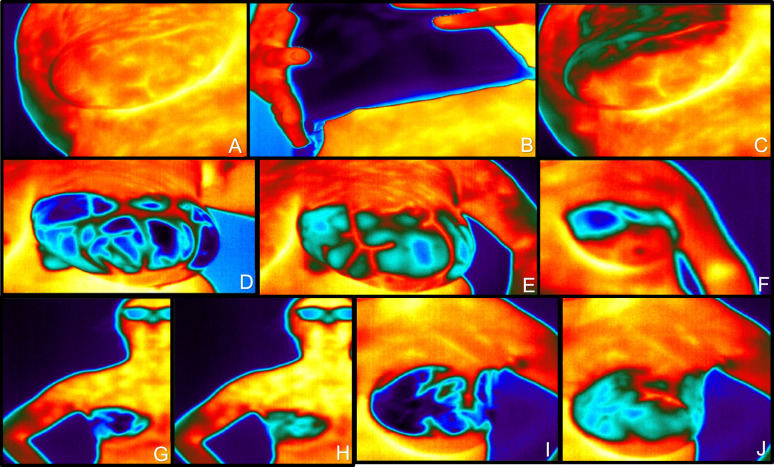
Top row: sequence of cooling with insufficient covering the breast (**A**–**C**); middle row, the left and middle image show cooling pad problems with the coverage and inhomogeneous cooling (**D**, **E**); the right imaging in the middle row shows to little cooling pad (**F**) for breast size and camera position to far away; the two left pictures of the lower row show camera size to far away (**G**, **H**) and the two right images on the lower row show problems with coverage and inhomogeneous cooling in patients with larger breast size without coverage on the lower to quadrants of the breast (**I**, **J**)

Thirty-nine patients with palpable breast lumps were recruited in this trial. Table 1 shows patient and tumor characteristics. Further excluded from the final analysis were 21 patients from part one of the trial, during which the method was established and the imaging routine was implemented (see Figs. 2 and 5). The reasons for excluding these patients from the final analyses were image acquisition problems resulting in poor image quality. Mostly, the distance between the camera and the breast was too far, or the angle between the camera and the lesion position was incorrect. Second, insufficient cooling took place, whether the pad was too small or the breast tissue where the lesion was located was not covered and no native image was made.

18 patients were part of phase two of the trial, during which the imaging procedure was conducted following the newly established standardized protocol. Furthermore, three patients were excluded from the analysis because both sonography and mammography yielded inconspicuous results and no biopsy was performed. In one of these cases, an intramammary lymph node was identified and further evaluated by MRI, which confirmed the benign nature of the finding (BI-RADS 2). In the remaining two cases, neither sonography nor mammography revealed any correlate to the clinical findings. Consequently, a follow-up examination was scheduled for 12 months later. Thermographic imaging also showed no abnormalities in these cases, supporting the interpretation of true negative findings but without the knowledge of histology. Finally, 15 patients were evaluated, and the results from mammography and sonography were compared to HFI results regarding the position of the lesion and whether the lesion was detectable.

For the evaluation the breast anatomy was divided into four quadrants. HFI was considered positive if it could detect a lesion in the same quadrant as identified by mammography. HFI successfully detected six carcinomas when image quality was adequate. In one case, the incorrect quadrant was noted due to the large size of the breast. Overall, HFI detected 7 out of 11 (64%) carcinomas. One DCIS and three carcinomas were not detected due to insufficient cooling in the region of interest or improper positioning of the breast, which caused the camera to capture only the inner half of the breast. Three cases of fibrous mastopathy and one case of flat epithelial atypia were not detected correctly using the heat flow imaging.

Table 2 shows the patients where the HFI did not find the corresponding lesions. We did not calculate any sensitivity or specificity because of the feasibility character of the study.

**Table 2:** False negative findings of in heat flow imaging

Number	Final histology	BI-RADS	ACR	Position and size in MG	Position in HFI	Reason for discrepancy
1	NST and DCIS	5	B	UUQ, 40*40 mm	no lesion	Upper outer region was not seen in imaging, the patient rotated outwards, outer side of the breast not visible
2	DCIS	4	B	UOQ, 40*20 mm	No lesion	Cooling pad dislocated, did not cover the upper breast area
3	NST	5	D	LIQ, 22*11 mm	No lesion	upper central region was not cooled
4	NST	4	B	Retro-mammillary, 19*15 mm	No lesion	No cooling retromammillary, because of large breast with ptosis III°

*BI-RADS* breast imaging reporting and data system, *ACR* American college of radiology (Scale from a-d, normal tissue to extremely dense breast tissue); *DCIS* ductal carcinoma in situ; *MG* mammography; *HFI* heat flow imaging; *NST* no special type; *UOQ* upper outer quadrant; *UIQ* upper inner quadrant; *LIQ* lower inner quadrant

### Pain questionnaire evaluation

Following the HFI examination, 33 out of 39 (85%) patients fully completed a questionnaire detailing their pain experience during the procedure. Pain was assessed using a numerical rating scale ranging from zero to ten, where ten represents the maximum level of pain. The mean pain score reported was 0.7 (Median 0; SD 1.3). Participants were also queried about the presence of skin redness post-examination, which three (9%) patients reported. However, the redness resolved immediately after the procedure.

The patients were further asked to compare the pain experienced during the HFI to that of conventional mammography and sonography. Three (9%) patients reported that the pain was comparable to that of mammography, while the remaining (*N* = 30) patients indicated that the pain was less. Similarly, in comparison to sonography, three (9%) patients found the pain to be comparable, and all others reported less pain.

## Discussion

This prospective, single-center study evaluated the feasibility of HFI for detecting palpable breast lesions during routine outpatient consultations at a certified breast cancer center in Germany. The study employed a two-phase design: initially standardizing the imaging process to improve quality, followed by evaluating its potential to detect palpable breast lesions in the correct anatomical location. The results suggest that HFI can identify palpable lesions under specific conditions, supporting its potential as a supplementary diagnostic tool for patients presenting with palpable breast lesions, but still is a diagnostic imaging method only recommended to use in clinical trials.

The approach in this study differs from prior research in its dynamic imaging methodology, recording video frames rather than static images. Previous studies, such as those using digital infrared thermal imaging (DITI), typically employed three imaging positions (left oblique, anterior, and right oblique) for static captures, enabling a broader perspective of breast anatomy [33]. The dynamic aspect of our protocol may enhance lesion detectability by capturing temporal changes in heat distribution, though this requires further validation against established methods.

Another important factor of the imaging process is the cooling. Barros et al. evaluated if different stages and depths of hypothermia can improve early detection of breast cancer in HFI. They used three steps of the procedure, stationary, hypothermy and recovery, whether the cooling process was evaluated with 0, 5, 10 and 15 °C up to 20 min. They could show that for intermediately deep lying tumors cooled to 0 °C, the increase in thermal contrast was 72%, and cooling to 15 °C was 36% [34]. In comparison, our study used a less intensive cooling protocol (8 °C for 30 s), which might have limited the thermal contrast observed, particularly for deep-seated lesions. Lesions close to the pectoral muscle will need more precise workup because they will be hardest to address with the cooling and reheating process.

The results of the study of Barros showed that the reduction of the glandular tissue which takes place in older especially in postmenopausal women causes a decrease in the thermal contrast, which is underlying the hypothesis that in younger patients HFI could be more precise [34]. The age distribution in our collective was equal between pre- and postmenopausal women, but imaging quality did not significantly differ between those groups, which might be due to sample size.

Computational analyses suggest that shorter cooling durations yield maximum thermal contrast early in the recovery phase, while prolonged cooling results in broader, less pronounced thermal peaks [35]. These findings underscore the need to optimize cooling protocols for future studies. We applied a shorter cooling protocol which is easier to perform in a real-world setting, which still needs, however, to be examined in larger studies, to control if a less intensive cooling process has a negative effect on diagnostic outcome. We could show that if the cooling area is large enough and the coverage of the breast tissue is uniformly, the imaging quality is satisfactory.

Studies utilizing advanced automatic segmentation methods have demonstrated promising results in HFI. For instance, one study using a specific dataset reported a sensitivity of 98.8%, specificity of 98.4%, and an overall accuracy of 93.1% [36]. Advances in automated segmentation and artificial intelligence (AI)-driven analysis continues to enhance the diagnostic accuracy of HFI. Studies employing convolutional and recurrent neural networks, such as the VGG16-LSTM model, have achieved impressive diagnostic metrics, with sensitivity, specificity, and accuracy exceeding 90% in some cases [37]. Comparative studies provide additional context for thermography's performance. For instance, in literature comparable sensitivity between mammography (80.5%) and thermography (81.6%) is reported, though specificity was higher for mammography (73.3% vs. 57.8%) [26]. In other studies, thermography identified 58 of 60 malignancies, with 97% sensitivity and 44% specificity, and 82% negative predictive value [30], or by using a dynamic computerized infrared imaging system, in patients where breast biopsy was recommended, 97% sensitivity, 14% specificity, a 95% negative predictive value, and a 24% positive predictive value [27]. These findings highlight t HFI’s strengths in detecting abnormalities while emphasizing the need for improved specificity. We did not calculate sensitivity and specificity because of the feasibility character of the trial but future research is needed in larger prospective multicenter trials to find the right indication for HFI.

An important aspect for patients with HFI is that it is a save method to perform and also the pain questionnaire we conducted showed only minimal pain from the cooling process compared to sonography and mammography. Further key aspects are the time component and radiation exposure, imaging using HFI takes 90 s in total including the cooling time, which is a time saving procedure. In addition and no radiation is needed.

### Limitations

Despite promising results, this study has several limitations. First, the sample size was relatively small and limited to a single center, restricting the generalizability of the findings. The quantitative metrics such as sensitivity and specificity were not calculated due to the study's feasibility focus, necessitating larger multicenter trials for robust validation. Furthermore, external factors such as ambient temperature and individual variations in skin blood flow may influence HFI outcomes, underscoring the need for controlled environments and standardized protocols.

Furthermore, the imaging method is hard to establish in a routine setting and an automated reproductive imaging tool is necessary to produce reliable data and machine learning could improve reading performance. HFI is not meant to substitute mammography which was declared by the U.S. Food and Drug Administration (FDA) in 2023 but was declared as a “adjunctive” tool [38]. In our trial we did not aim to substitute any existing imaging method as mammography or sonography or to implement a new screening tool. This study focused on testing HFI as diagnostic tool and the use in daily routines.

## Conclusion

The findings of this study suggest that dynamic HFI could be a promising, non-invasive complement to mammography, particularly for younger women or those with dense breast tissue, actually tested as additional diagnostic tool for patients with palpable breast lesions. However, further research in clinical trials with larger patient groups and optimized imaging protocols is needed to confirm the diagnostic accuracy of this method and define the correct indication.

## Supplementary Information

Below is the link to the electronic supplementary material.

Supplementary file1 (DOCX 56 KB)

## Data Availability

No datasets were generated or analysed during the current study.
