# Supplementary material for: Evaluation and feasibility of diagnostic heatflow imaging in patients with palpable breast lesions: a pilot study
Source: Arch Gynecol Obstet. 2025 Jun 28;312(4):1107–15. doi: 10.1007/s00404-025-08093-5 (PMC12414028; doi:10.1007/s00404-025-08093-5)
Supplement: Supplementary file 1 — Supplementary file1 (DOCX 56 KB) [file 404_2025_8093_MOESM1_ESM.docx]

**Supplementary Materials**

**Pain Questionnaire**

**Supplement Table 1**

|  | Strongly agree | Agree | Neutral | Rather disagree | Disagree | Not applicable |
| --- | --- | --- | --- | --- | --- | --- |
| Pain |  |  |  |  |  |  |
| I experienced pain in my breast during the examination |  |  |  |  |  |  |
| I found the cold unpleasant |  |  |  |  |  |  |
| I experienced pain in other parts of my body during the examination |  |  |  |  |  |  |
| The pain ended with the examination |  |  | | |  |  |
| Skin Redness |  |  |  |  |  |  |
| I had redness on my breast after the examination |  |  |  |  |  |  |
| I had redness on other parts of my body after the examination |  |  |  |  |  |  |
| The redness disappeared after the examination |  |  | | |  |  |
| Comparison with Mammography |  |  |  |  |  |  |
| The pain is comparable to that of a mammography |  |  |  |  |  |  |
| The pain is stronger than that of a mammography |  |  |  |  |  |  |
| The mammography examination is shorter than the heat flow imaging examination |  |  |  |  |  |  |
| Comparison with Ultrasound |  |  |  |  |  |  |
| The pain is comparable to that of an ultrasound |  |  |  |  |  |  |
| The pain is stronger than that of an ultrasound |  |  |  |  |  |  |
| The ultrasound examination is shorter than the heat flow imaging examination |  |  |  |  |  |  |
| Overall Assessment of Pain During the Examination | 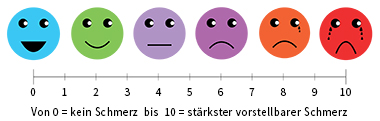 | | | | | |
| To be completed by the physician/examiner |  |  |  |  |  |  |
| Visible redness of the examined breast? |  |  |  |  |  |  |
| Visible hematoma on the examined breast? |  |  |  |  |  |  |
| Visible bleeding on the examined breast? |  |  |  |  |  |  |
| Other signs of bruising/injury visible? |  |  |  |  |  |  |
| Additional comments: | | | | | |  |
